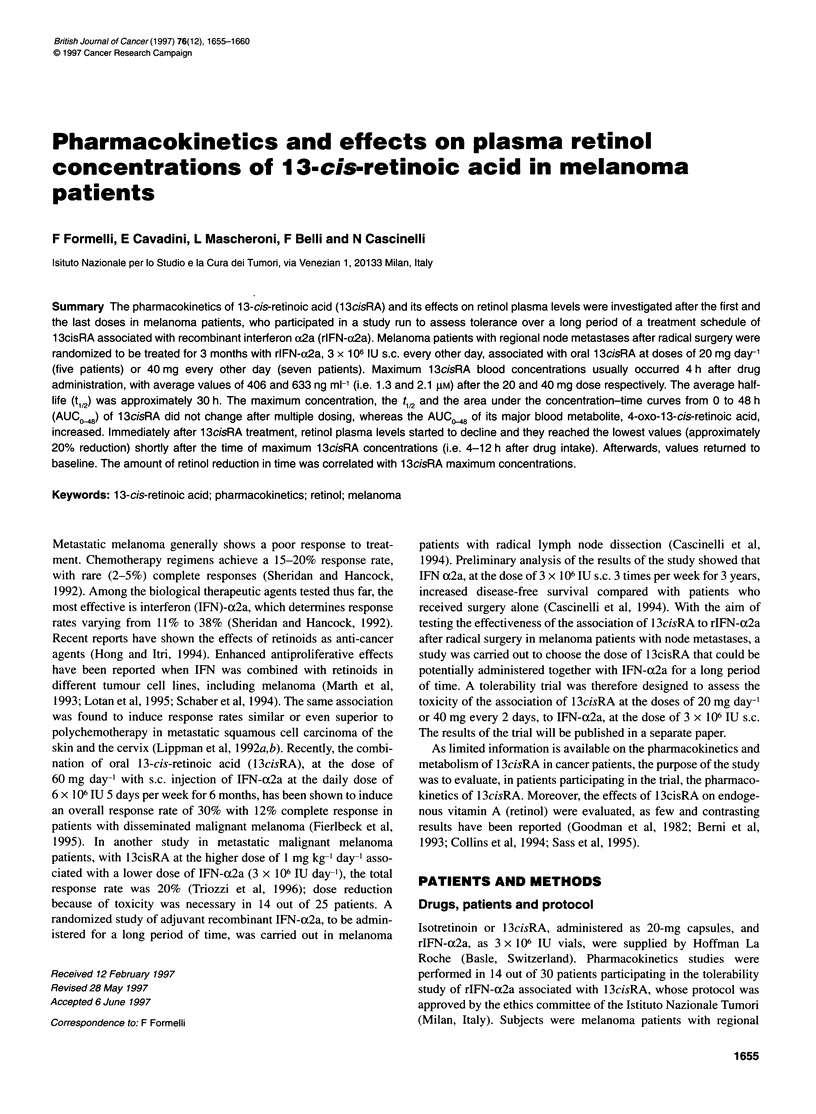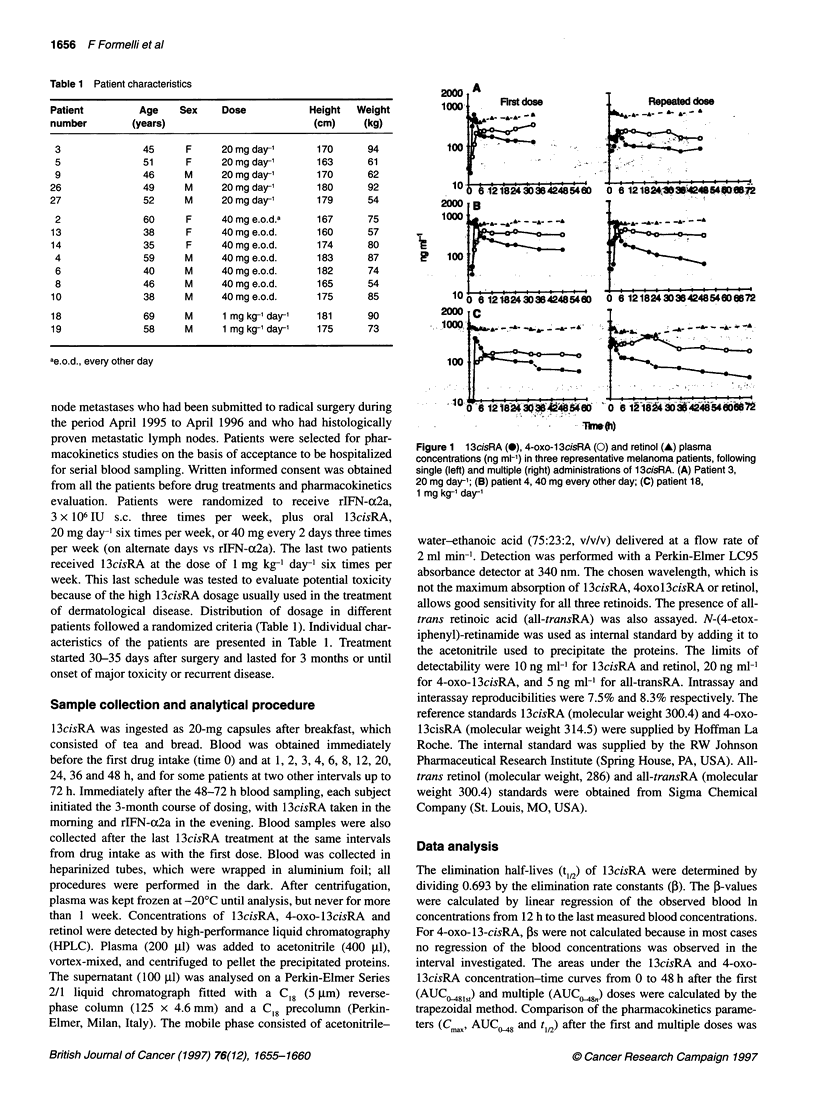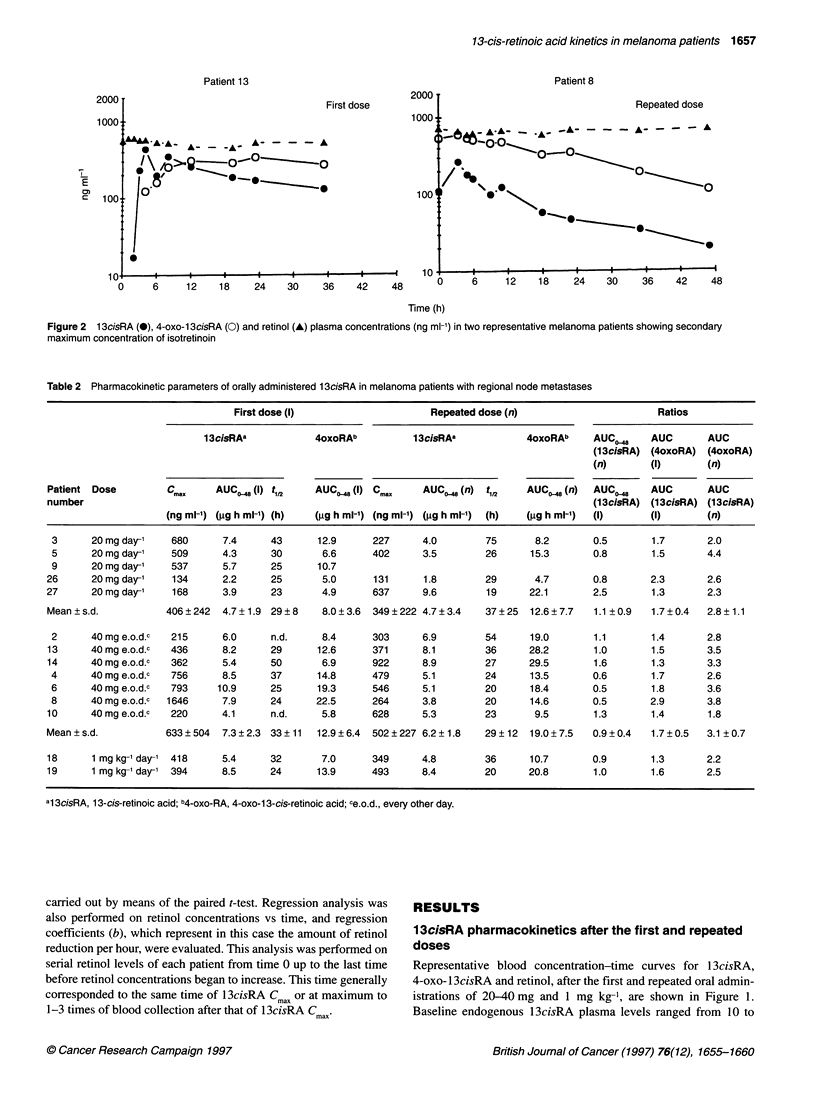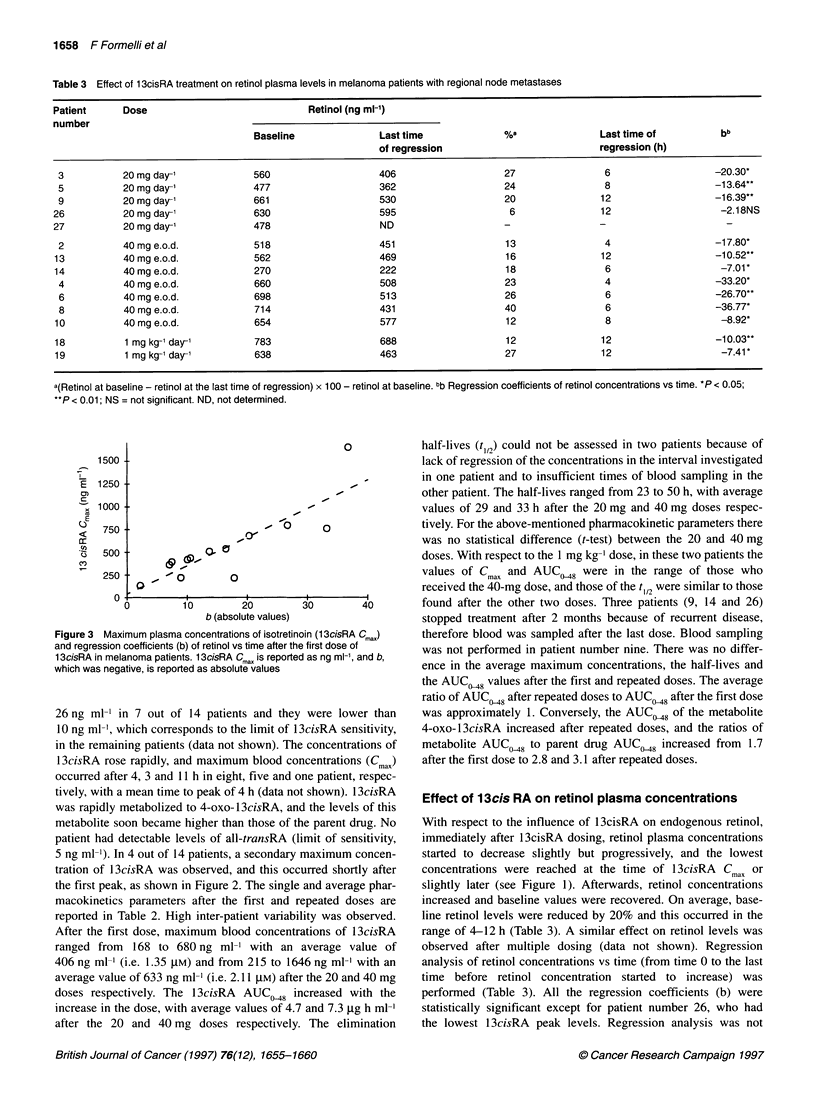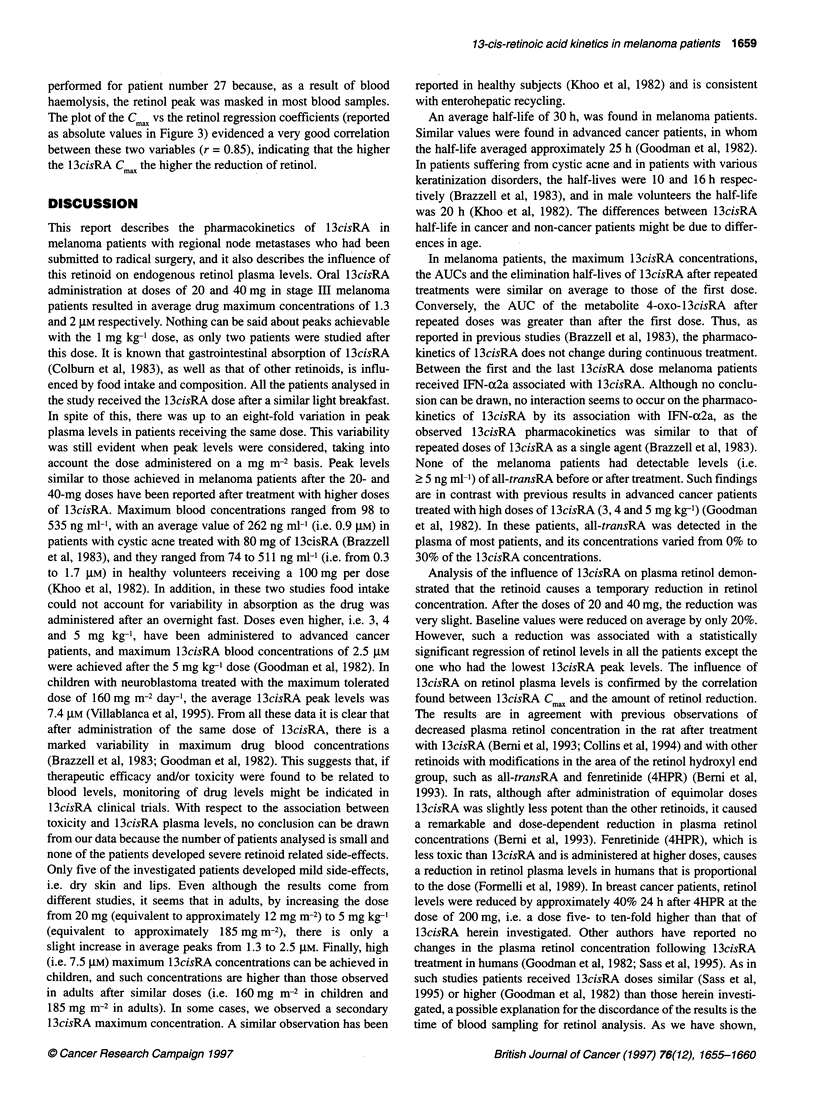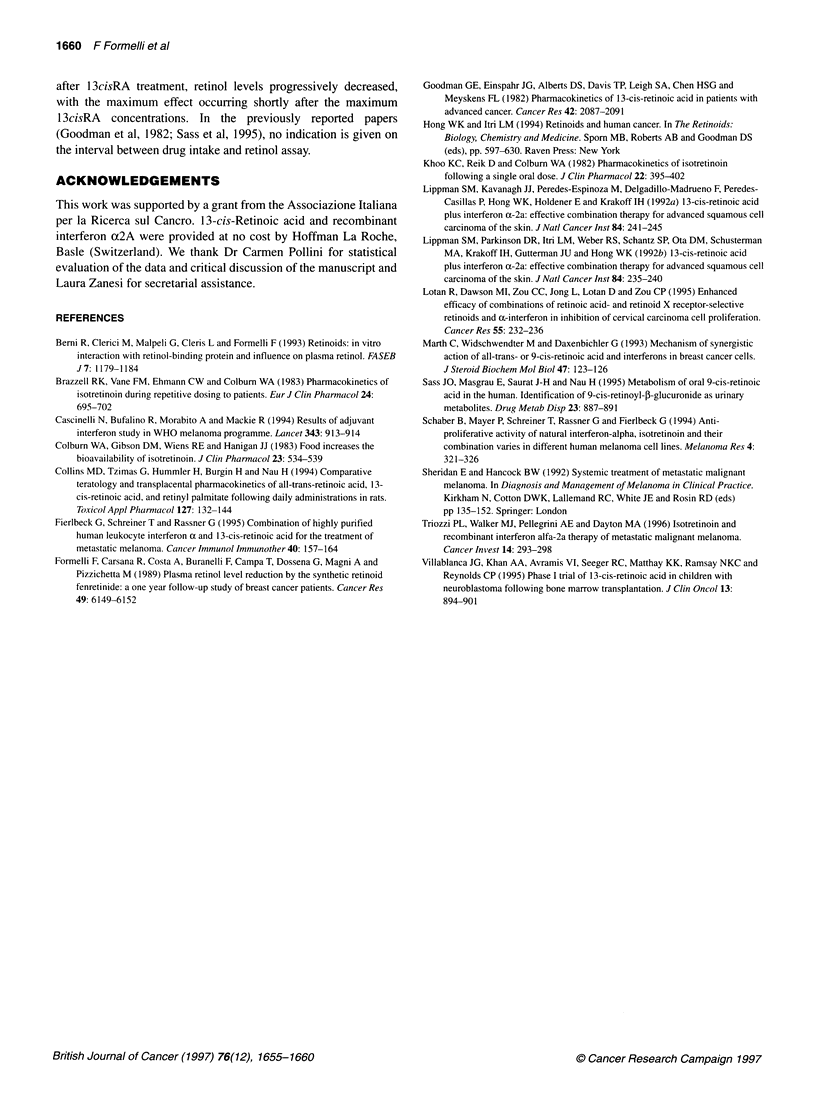# Pharmacokinetics and effects on plasma retinol concentrations of 13-cis-retinoic acid in melanoma patients.

**DOI:** 10.1038/bjc.1997.613

**Published:** 1997

**Authors:** F. Formelli, E. Cavadini, L. Mascheroni, F. Belli, N. Cascinelli

**Affiliations:** Istituto Nazionale per lo Studio e la Cura dei Tumori, Milan, Italy.

## Abstract

The pharmacokinetics of 13-cis-retinoic acid (13cisRA) and its effects on retinol plasma levels were investigated after the first and the last doses in melanoma patients, who participated in a study run to assess tolerance over a long period of a treatment schedule of 13cisRA associated with recombinant interferon alpha2a (rIFN-alpha2a). Melanoma patients with regional node metastases after radical surgery were randomized to be treated for 3 months with rIFN-alpha2a, 3 x 10(6) IU s.c. every other day, associated with oral 13cisRA at doses of 20 mg day(-1) (five patients) or 40 mg every other day (seven patients). Maximum 13cisRA blood concentrations usually occurred 4 h after drug administration, with average values of 406 and 633 ng ml(-1) (i.e. 1.3 and 2.1 microM) after the 20 and 40 mg dose respectively. The average half-life (t(1/2)) was approximately 30 h. The maximum concentration, the t(1/2) and the area under the concentration-time curves from 0 to 48 h (AUC(0-48)) of 13cisRA did not change after multiple dosing, whereas the AUC(0-48) of its major blood metabolite, 4-oxo-13-cis-retinoic acid, increased. Immediately after 13cisRA treatment, retinol plasma levels started to decline and they reached the lowest values (approximately 20% reduction) shortly after the time of maximum 13cisRA concentrations (i.e. 4-12 h after drug intake). Afterwards, values returned to baseline. The amount of retinol reduction in time was correlated with 13cisRA maximum concentrations.


*British Journal of Cancer* (1997) **76**(12), 1655–1660
© 1997 Cancer Research Campaign

### Pharmacokinetics and effects on plasma retinol concentrations of 13-*cis*-retinoic acid in melanoma patients

**F Formelli, E Cavadini, L Mascheroni, F Belli and N Cascinelli**

Isituto Nazionale per lo Studio e la Cura dei Tumori, via Venezian 1, 20133 Milan, Italy

**Summary** The pharmacokinetics of 13-*cis*-retinoic acid (13*cis*RA) and its effects on retinol plasma levels were investigated after the first and the last doses in melanoma patients, who participated in a study run to assess tolerance over a long period of a treatment schedule of 13cisRA associated with recombinant interferon α2a (rIFN-α2a). Melanoma patients with regional node metastases after radical surgery were randomized to be treated for 3 months with rIFN-α2a, 3 × 10[6] IU s.c. every other day, associated with oral 13*cis*RA at doses of 20 mg day$^{-1}$ (five patients) or 40 mg every other day (seven patients). Maximum 13*cis*RA blood concentrations usually occurred 4 h after drug administration, with average values of 406 and 633 ng ml$^{-1}$ (i.e. 1.3 and 2.1 μM) after the 20 and 40 mg dose respectively. The average half-life ($t_{1/2}$) was approximately 30 h. The maximum concentration, the $t_{1/2}$ and the area under the concentration–time curves from 0 to 48 h ($AUC_{0–48}$) of 13*cis*RA did not change after multiple dosing, whereas the $AUC_{0–48}$ of its major blood metabolite, 4-oxo-13-*cis*-retinoic acid, increased. Immediately after 13*cis*RA treatment, retinol plasma levels started to decline and they reached the lowest values (approximately 20% reduction) shortly after the time of maximum 13*cis*RA concentrations (i.e. 4–12 h after drug intake). Afterwards, values returned to baseline. The amount of retinol reduction in time was correlated with 13*cis*RA maximum concentrations.

**Keywords:** 13-*cis*-retinoic acid; pharmacokinetics; retinol; melanoma

Metastatic melanoma generally shows a poor response to treatment. Chemotherapy regimens achieve a 15–20% response rate, with rare (2–5%) complete responses (Sheridan and Hancock, 1992). Among the biological therapeutic agents tested thus far, the most effective is interferon (IFN)-α2a, which determines response rates varying from 11% to 38% (Sheridan and Hancock, 1992). Recent reports have shown the effects of retinoids as anti-cancer agents (Hong and Itri, 1994). Enhanced antiproliferative effects have been reported when IFN was combined with retinoids in different tumour cell lines, including melanoma (Marth et al, 1993; Lotan et al, 1995; Schaber et al, 1994). The same association was found to induce response rates similar or even superior to polychemotherapy in metastatic squamous cell carcinoma of the skin and the cervix (Lippman et al, 1992*a,b*). Recently, the combination of oral 13-*cis*-retinoic acid (13*cis*RA), at the dose of 60 mg day$^{-1}$ with s.c. injection of IFN-α2a at the daily dose of 6 × 10[6] IU 5 days per week for 6 months, has been shown to induce an overall response rate of 30% with 12% complete response in patients with disseminated malignant melanoma (Fierlbeck et al, 1995). In another study in metastatic malignant melanoma patients, with 13cisRA at the higher dose of 1 mg kg$^{-1}$ day$^{-1}$ associated with a lower dose of IFN-α2a (3 × 10[6] IU day$^{-1}$), the total response rate was 20% (Triozzi et al, 1996); dose reduction because of toxicity was necessary in 14 out of 25 patients. A randomized study of adjuvant recombinant IFN-α2a, to be administered for a long period of time, was carried out in melanoma patients with radical lymph node dissection (Cascinelli et al, 1994). Preliminary analysis of the results of the study showed that IFN α2a, at the dose of 3 × 10[6] IU s.c. 3 times per week for 3 years, increased disease-free survival compared with patients who received surgery alone (Cascinelli et al, 1994). With the aim of testing the effectiveness of the association of 13*cis*RA to rIFN-α2a after radical surgery in melanoma patients with node metastases, a study was carried out to choose the dose of 13cisRA that could be potentially administered together with IFN-α2a for a long period of time. A tolerability trial was therefore designed to assess the toxicity of the association of 13*cis*RA at the doses of 20 mg day$^{-1}$ or 40 mg every 2 days, to IFN-α2a, at the dose of 3 × 10[6] IU s.c. The results of the trial will be published in a separate paper.

As limited information is available on the pharmacokinetics and metabolism of 13*cis*RA in cancer patients, the purpose of the study was to evaluate, in patients participating in the trial, the pharmacokinetics of 13*cis*RA. Moreover, the effects of 13cisRA on endogenous vitamin A (retinol) were evaluated, as few and contrasting results have been reported (Goodman et al, 1982; Berni et al, 1993; Collins et al, 1994; Sass et al, 1995).

*Received 12 February 1997*
*Revised 28 May 1997*
*Accepted 6 June 1997*

*Correspondence to:* F Formelli

### PATIENTS AND METHODS

### Drugs, patients and protocol

Isotretinoin or 13*cis*RA, administered as 20-mg capsules, and rIFN-α2a, as 3 × 10[6] IU vials, were supplied by Hoffman La Roche (Basle, Switzerland). Pharmacokinetics studies were performed in 14 out of 30 patients participating in the tolerability study of rIFN-α2a associated with 13*cis*RA, whose protocol was approved by the ethics committee of the Istituto Nazionale Tumori (Milan, Italy). Subjects were melanoma patients with regional

**Table 1** Patient characteristics

| Patient number | Age (years) | Sex | Dose | Height (cm) | Weight (kg) |
|---|---|---|---|---|---|
| 3 | 45 | F | 20 mg day$^{-1}$ | 170 | 94 |
| 5 | 51 | F | 20 mg day$^{-1}$ | 163 | 61 |
| 9 | 46 | M | 20 mg day$^{-1}$ | 170 | 62 |
| 26 | 49 | M | 20 mg day$^{-1}$ | 180 | 92 |
| 27 | 52 | M | 20 mg day$^{-1}$ | 179 | 54 |
| 2 | 60 | F | 40 mg e.o.d.[a] | 167 | 75 |
| 13 | 38 | F | 40 mg e.o.d. | 160 | 57 |
| 14 | 35 | F | 40 mg e.o.d. | 174 | 80 |
| 4 | 59 | M | 40 mg e.o.d. | 183 | 87 |
| 6 | 40 | M | 40 mg e.o.d. | 182 | 74 |
| 8 | 46 | M | 40 mg e.o.d. | 165 | 54 |
| 10 | 38 | M | 40 mg e.o.d. | 175 | 85 |
| 18 | 69 | M | 1 mg kg$^{-1}$ day$^{-1}$ | 181 | 90 |
| 19 | 58 | M | 1 mg kg$^{-1}$ day$^{-1}$ | 175 | 73 |

[a]e.o.d., every other day

**Figure 1** 13*cis*RA (●), 4-oxo-13*cis*RA (○) and retinol (▲) plasma concentrations (ng ml$^{-1}$) in three representative melanoma patients, following single (left) and multiple (right) administrations of 13*cis*RA. (**A**) Patient 3, 20 mg day$^{-1}$; (**B**) patient 4, 40 mg every other day; (**C**) patient 18, 1 mg kg$^{-1}$ day$^{-1}$

node metastases who had been submitted to radical surgery during the period April 1995 to April 1996 and who had histologically proven metastatic lymph nodes. Patients were selected for pharmacokinetics studies on the basis of acceptance to be hospitalized for serial blood sampling. Written informed consent was obtained from all the patients before drug treatments and pharmacokinetics evaluation. Patients were randomized to receive rIFN-α2a, $3 \times 10^6$ IU s.c. three times per week, plus oral 13*cis*RA, 20 mg day$^{-1}$ six times per week, or 40 mg every 2 days three times per week (on alternate days vs rIFN-α2a). The last two patients received 13*cis*RA at the dose of 1 mg kg$^{-1}$ day$^{-1}$ six times per week. This last schedule was tested to evaluate potential toxicity because of the high 13*cis*RA dosage usually used in the treatment of dermatological disease. Distribution of dosage in different patients followed a randomized criteria (Table 1). Individual characteristics of the patients are presented in Table 1. Treatment started 30–35 days after surgery and lasted for 3 months or until onset of major toxicity or recurrent disease.

### Sample collection and analytical procedure

13*cis*RA was ingested as 20-mg capsules after breakfast, which consisted of tea and bread. Blood was obtained immediately before the first drug intake (time 0) and at 1, 2, 3, 4, 6, 8, 12, 20, 24, 36 and 48 h, and for some patients at two other intervals up to 72 h. Immediately after the 48–72 h blood sampling, each subject initiated the 3-month course of dosing, with 13*cis*RA taken in the morning and rIFN-α2a in the evening. Blood samples were also collected after the last 13*cis*RA treatment at the same intervals from drug intake as with the first dose. Blood was collected in heparinized tubes, which were wrapped in aluminium foil; all procedures were performed in the dark. After centrifugation, plasma was kept frozen at –20°C until analysis, but never for more than 1 week. Concentrations of 13*cis*RA, 4-oxo-13*cis*RA and retinol were detected by high-performance liquid chromatography (HPLC). Plasma (200 μl) was added to acetonitrile (400 μl), vortex-mixed, and centrifuged to pellet the precipitated proteins. The supernatant (100 μl) was analysed on a Perkin-Elmer Series 2/1 liquid chromatograph fitted with a $C_{18}$ (5 μm) reverse-phase column (125 × 4.6 mm) and a $C_{18}$ precolumn (Perkin-Elmer, Milan, Italy). The mobile phase consisted of acetonitrile–water–ethanoic acid (75:23:2, v/v/v) delivered at a flow rate of 2 ml min$^{-1}$. Detection was performed with a Perkin-Elmer LC95 absorbance detector at 340 nm. The chosen wavelength, which is not the maximum absorption of 13*cis*RA, 4oxo13*cis*RA or retinol, allows good sensitivity for all three retinoids. The presence of all-*trans* retinoic acid (all-*trans*RA) was also assayed. *N*-(4-etoxiphenyl)-retinamide was used as internal standard by adding it to the acetonitrile used to precipitate the proteins. The limits of detectability were 10 ng ml$^{-1}$ for 13*cis*RA and retinol, 20 ng ml$^{-1}$ for 4-oxo-13*cis*RA, and 5 ng ml$^{-1}$ for all-transRA. Intrassay and interassay reproducibilities were 7.5% and 8.3% respectively. The reference standards 13*cis*RA (molecular weight 300.4) and 4-oxo-13cisRA (molecular weight 314.5) were supplied by Hoffman La Roche. The internal standard was supplied by the RW Johnson Pharmaceutical Research Institute (Spring House, PA, USA). All-*trans* retinol (molecular weight, 286) and all-*trans*RA (molecular weight 300.4) standards were obtained from Sigma Chemical Company (St. Louis, MO, USA).

### Data analysis

The elimination half-lives ($t_{1/2}$) of 13*cis*RA were determined by dividing 0.693 by the elimination rate constants (β). The β-values were calculated by linear regression of the observed blood ln concentrations from 12 h to the last measured blood concentrations. For 4-oxo-13-*cis*RA, βs were not calculated because in most cases no regression of the blood concentrations was observed in the interval investigated. The areas under the 13*cis*RA and 4-oxo-13*cis*RA concentration–time curves from 0 to 48 h after the first ($AUC_{0–481st}$) and multiple ($AUC_{0–48n}$) doses were calculated by the trapezoidal method. Comparison of the pharmacokinetics parameters ($C_{max}$, $AUC_{0–48}$ and $t_{1/2}$) after the first and multiple doses was

*© Cancer Research Campaign 1997*

**Figure 2** 13*cis*RA (●), 4-oxo-13*cis*RA (○) and retinol (▲) plasma concentrations (ng ml$^{-1}$) in two representative melanoma patients showing secondary maximum concentration of isotretinoin

**Table 2** Pharmacokinetic parameters of orally administered 13*cis*RA in melanoma patients with regional node metastases

| | | **First dose (I)** | | | | **Repeated dose (*n*)** | | | | **Ratios** | | |
|---|---|---|---|---|---|---|---|---|---|---|---|---|
| | | **13*cis*RA**[a] | | | **4oxoRA**[b] | **13*cis*RA**[a] | | | **4oxoRA**[b] | **$AUC_{0-48}$ (13*cis*RA) (*n*)** | **AUC (4oxoRA) (I)** | **AUC (4oxoRA) (*n*)** |
| **Patient number** | **Dose** | **$C_{max}$ (ng ml$^{-1}$)** | **$AUC_{0-48}$ (I) (µg h ml$^{-1}$)** | **$t_{1/2}$ (h)** | **$AUC_{0-48}$ (I) (µg h ml$^{-1}$)** | **$C_{max}$ (ng ml$^{-1}$)** | **$AUC_{0-48}$ (*n*) (µg h ml$^{-1}$)** | **$t_{1/2}$ (h)** | **$AUC_{0-48}$ (*n*) (µg h ml$^{-1}$)** | **$AUC_{0-48}$ (13*cis*RA) (I)** | **AUC (13*cis*RA) (I)** | **AUC (13*cis*RA) (*n*)** |
| 3 | 20 mg day$^{-1}$ | 680 | 7.4 | 43 | 12.9 | 227 | 4.0 | 75 | 8.2 | 0.5 | 1.7 | 2.0 |
| 5 | 20 mg day$^{-1}$ | 509 | 4.3 | 30 | 6.6 | 402 | 3.5 | 26 | 15.3 | 0.8 | 1.5 | 4.4 |
| 9 | 20 mg day$^{-1}$ | 537 | 5.7 | 25 | 10.7 | | | | | | | |
| 26 | 20 mg day$^{-1}$ | 134 | 2.2 | 25 | 5.0 | 131 | 1.8 | 29 | 4.7 | 0.8 | 2.3 | 2.6 |
| 27 | 20 mg day$^{-1}$ | 168 | 3.9 | 23 | 4.9 | 637 | 9.6 | 19 | 22.1 | 2.5 | 1.3 | 2.3 |
| Mean ± s.d. | | 406 ± 242 | 4.7 ± 1.9 | 29 ± 8 | 8.0 ± 3.6 | 349 ± 222 | 4.7 ± 3.4 | 37 ± 25 | 12.6 ± 7.7 | 1.1 ± 0.9 | 1.7 ± 0.4 | 2.8 ± 1.1 |
| 2 | 40 mg e.o.d.[c] | 215 | 6.0 | n.d. | 8.4 | 303 | 6.9 | 54 | 19.0 | 1.1 | 1.4 | 2.8 |
| 13 | 40 mg e.o.d.[c] | 436 | 8.2 | 29 | 12.6 | 371 | 8.1 | 36 | 28.2 | 1.0 | 1.5 | 3.5 |
| 14 | 40 mg e.o.d.[c] | 362 | 5.4 | 50 | 6.9 | 922 | 8.9 | 27 | 29.5 | 1.6 | 1.3 | 3.3 |
| 4 | 40 mg e.o.d.[c] | 756 | 8.5 | 37 | 14.8 | 479 | 5.1 | 24 | 13.5 | 0.6 | 1.7 | 2.6 |
| 6 | 40 mg e.o.d.[c] | 793 | 10.9 | 25 | 19.3 | 546 | 5.1 | 20 | 18.4 | 0.5 | 1.8 | 3.6 |
| 8 | 40 mg e.o.d.[c] | 1646 | 7.9 | 24 | 22.5 | 264 | 3.8 | 20 | 14.6 | 0.5 | 2.9 | 3.8 |
| 10 | 40 mg e.o.d.[c] | 220 | 4.1 | n.d. | 5.8 | 628 | 5.3 | 23 | 9.5 | 1.3 | 1.4 | 1.8 |
| Mean ± s.d. | | 633 ± 504 | 7.3 ± 2.3 | 33 ± 11 | 12.9 ± 6.4 | 502 ± 227 | 6.2 ± 1.8 | 29 ± 12 | 19.0 ± 7.5 | 0.9 ± 0.4 | 1.7 ± 0.5 | 3.1 ± 0.7 |
| 18 | 1 mg kg$^{-1}$ day$^{-1}$ | 418 | 5.4 | 32 | 7.0 | 349 | 4.8 | 36 | 10.7 | 0.9 | 1.3 | 2.2 |
| 19 | 1 mg kg$^{-1}$ day$^{-1}$ | 394 | 8.5 | 24 | 13.9 | 493 | 8.4 | 20 | 20.8 | 1.0 | 1.6 | 2.5 |

[a]13*cis*RA, 13-*cis*-retinoic acid; [b]4-oxo-RA, 4-oxo-13-*cis*-retinoic acid; [c]e.o.d., every other day.

carried out by means of the paired *t*-test. Regression analysis was also performed on retinol concentrations vs time, and regression coefficients (*b*), which represent in this case the amount of retinol reduction per hour, were evaluated. This analysis was performed on serial retinol levels of each patient from time 0 up to the last time before retinol concentrations began to increase. This time generally corresponded to the same time of 13*cis*RA $C_{max}$ or at maximum to 1–3 times of blood collection after that of 13*cis*RA $C_{max}$.

### RESULTS

### 13*cis*RA pharmacokinetics after the first and repeated doses

Representative blood concentration–time curves for 13*cis*RA, 4-oxo-13*cis*RA and retinol, after the first and repeated oral administrations of 20–40 mg and 1 mg kg$^{-1}$, are shown in Figure 1. Baseline endogenous 13*cis*RA plasma levels ranged from 10 to

© Cancer Research Campaign 1997 

Table 3 Effect of 13cisRA treatment on retinol plasma levels in melanoma patients with regional node metastases

| Patient number | Dose | Retinol ($ng\ ml^{-1}$) | | %[a] | Last time of regression (h) | b[b] |
|---|---|---|---|---|---|---|
| | | Baseline | Last time of regression | | | |
| 3 | 20 mg $day^{-1}$ | 560 | 406 | 27 | 6 | –20.30* |
| 5 | 20 mg $day^{-1}$ | 477 | 362 | 24 | 8 | –13.64** |
| 9 | 20 mg $day^{-1}$ | 661 | 530 | 20 | 12 | –16.39** |
| 26 | 20 mg $day^{-1}$ | 630 | 595 | 6 | 12 | –2.18NS |
| 27 | 20 mg $day^{-1}$ | 478 | ND | – | – | – |
| 2 | 40 mg e.o.d. | 518 | 451 | 13 | 4 | –17.80* |
| 13 | 40 mg e.o.d. | 562 | 469 | 16 | 12 | –10.52** |
| 14 | 40 mg e.o.d. | 270 | 222 | 18 | 6 | –7.01* |
| 4 | 40 mg e.o.d. | 660 | 508 | 23 | 4 | –33.20* |
| 6 | 40 mg e.o.d. | 698 | 513 | 26 | 6 | –26.70** |
| 8 | 40 mg e.o.d. | 714 | 431 | 40 | 6 | –36.77* |
| 10 | 40 mg e.o.d. | 654 | 577 | 12 | 8 | –8.92* |
| 18 | 1 mg $kg^{-1}$ $day^{-1}$ | 783 | 688 | 12 | 12 | –10.03** |
| 19 | 1 mg $kg^{-1}$ $day^{-1}$ | 638 | 463 | 27 | 12 | –7.41* |

[a](Retinol at baseline – retinol at the last time of regression) × 100 – retinol at baseline. [b]b Regression coefficients of retinol concentrations vs time. *$P < 0.05$; **$P < 0.01$; NS = not significant. ND, not determined.

Figure 3 Maximum plasma concentrations of isotretinoin (13*cis*RA $C_{max}$) and regression coefficients (b) of retinol vs time after the first dose of 13*cis*RA in melanoma patients. 13*cis*RA $C_{max}$ is reported as ng $ml^{-1}$, and *b*, which was negative, is reported as absolute values

26 ng $ml^{-1}$ in 7 out of 14 patients and they were lower than 10 ng $ml^{-1}$, which corresponds to the limit of 13*cis*RA sensitivity, in the remaining patients (data not shown). The concentrations of 13*cis*RA rose rapidly, and maximum blood concentrations ($C_{max}$) occurred after 4, 3 and 11 h in eight, five and one patient, respectively, with a mean time to peak of 4 h (data not shown). 13*cis*RA was rapidly metabolized to 4-oxo-13*cis*RA, and the levels of this metabolite soon became higher than those of the parent drug. No patient had detectable levels of all-*trans*RA (limit of sensitivity, 5 ng $ml^{-1}$). In 4 out of 14 patients, a secondary maximum concentration of 13*cis*RA was observed, and this occurred shortly after the first peak, as shown in Figure 2. The single and average pharmacokinetics parameters after the first and repeated doses are reported in Table 2. High inter-patient variability was observed. After the first dose, maximum blood concentrations of 13*cis*RA ranged from 168 to 680 ng $ml^{-1}$ with an average value of 406 ng $ml^{-1}$ (i.e. 1.35 μM) and from 215 to 1646 ng $ml^{-1}$ with an average value of 633 ng $ml^{-1}$ (i.e. 2.11 μM) after the 20 and 40 mg doses respectively. The 13*cis*RA $AUC_{0-48}$ increased with the increase in the dose, with average values of 4.7 and 7.3 μg h $ml^{-1}$ after the 20 and 40 mg doses respectively. The elimination half-lives ($t_{1/2}$) could not be assessed in two patients because of lack of regression of the concentrations in the interval investigated in one patient and to insufficient times of blood sampling in the other patient. The half-lives ranged from 23 to 50 h, with average values of 29 and 33 h after the 20 mg and 40 mg doses respectively. For the above-mentioned pharmacokinetic parameters there was no statistical difference (*t*-test) between the 20 and 40 mg doses. With respect to the 1 mg $kg^{-1}$ dose, in these two patients the values of $C_{max}$ and $AUC_{0-48}$ were in the range of those who received the 40-mg dose, and those of the $t_{1/2}$ were similar to those found after the other two doses. Three patients (9, 14 and 26) stopped treatment after 2 months because of recurrent disease, therefore blood was sampled after the last dose. Blood sampling was not performed in patient number nine. There was no difference in the average maximum concentrations, the half-lives and the $AUC_{0-48}$ values after the first and repeated doses. The average ratio of $AUC_{0-48}$ after repeated doses to $AUC_{0-48}$ after the first dose was approximately 1. Conversely, the $AUC_{0-48}$ of the metabolite 4-oxo-13*cis*RA increased after repeated doses, and the ratios of metabolite $AUC_{0-48}$ to parent drug $AUC_{0-48}$ increased from 1.7 after the first dose to 2.8 and 3.1 after repeated doses.

### Effect of 13*cis* RA on retinol plasma concentrations

With respect to the influence of 13cisRA on endogenous retinol, immediately after 13cisRA dosing, retinol plasma concentrations started to decrease slightly but progressively, and the lowest concentrations were reached at the time of 13*cis*RA $C_{max}$ or slightly later (see Figure 1). Afterwards, retinol concentrations increased and baseline values were recovered. On average, baseline retinol levels were reduced by 20% and this occurred in the range of 4–12 h (Table 3). A similar effect on retinol levels was observed after multiple dosing (data not shown). Regression analysis of retinol concentrations vs time (from time 0 to the last time before retinol concentration started to increase) was performed (Table 3). All the regression coefficients (b) were statistically significant except for patient number 26, who had the lowest 13*cis*RA peak levels. Regression analysis was not

 © Cancer Research Campaign 1997

performed for patient number 27 because, as a result of blood haemolysis, the retinol peak was masked in most blood samples. The plot of the $C_{max}$ vs the retinol regression coefficients (reported as absolute values in Figure 3) evidenced a very good correlation between these two variables ($r = 0.85$), indicating that the higher the 13*cis*RA $C_{max}$ the higher the reduction of retinol.

### DISCUSSION

This report describes the pharmacokinetics of 13*cis*RA in melanoma patients with regional node metastases who had been submitted to radical surgery, and it also describes the influence of this retinoid on endogenous retinol plasma levels. Oral 13*cis*RA administration at doses of 20 and 40 mg in stage III melanoma patients resulted in average drug maximum concentrations of 1.3 and 2 μM respectively. Nothing can be said about peaks achievable with the 1 mg $kg^{-1}$ dose, as only two patients were studied after this dose. It is known that gastrointestinal absorption of 13*cis*RA (Colburn et al, 1983), as well as that of other retinoids, is influenced by food intake and composition. All the patients analysed in the study received the 13*cis*RA dose after a similar light breakfast. In spite of this, there was up to an eight-fold variation in peak plasma levels in patients receiving the same dose. This variability was still evident when peak levels were considered, taking into account the dose administered on a mg $m^{-2}$ basis. Peak levels similar to those achieved in melanoma patients after the 20- and 40-mg doses have been reported after treatment with higher doses of 13*cis*RA. Maximum blood concentrations ranged from 98 to 535 ng $ml^{-1}$, with an average value of 262 ng $ml^{-1}$ (i.e. 0.9 μM) in patients with cystic acne treated with 80 mg of 13cisRA (Brazzell et al, 1983), and they ranged from 74 to 511 ng $ml^{-1}$ (i.e. from 0.3 to 1.7 μM) in healthy volunteers receiving a 100 mg per dose (Khoo et al, 1982). In addition, in these two studies food intake could not account for variability in absorption as the drug was administered after an overnight fast. Doses even higher, i.e. 3, 4 and 5 mg $kg^{-1}$, have been administered to advanced cancer patients, and maximum 13*cis*RA blood concentrations of 2.5 μM were achieved after the 5 mg $kg^{-1}$ dose (Goodman et al, 1982). In children with neuroblastoma treated with the maximum tolerated dose of 160 mg $m^{-2}$ $day^{-1}$, the average 13*cis*RA peak levels was 7.4 μM (Villablanca et al, 1995). From all these data it is clear that after administration of the same dose of 13*cis*RA, there is a marked variability in maximum drug blood concentrations (Brazzell et al, 1983; Goodman et al, 1982). This suggests that, if therapeutic efficacy and/or toxicity were found to be related to blood levels, monitoring of drug levels might be indicated in 13*cis*RA clinical trials. With respect to the association between toxicity and 13*cis*RA plasma levels, no conclusion can be drawn from our data because the number of patients analysed is small and none of the patients developed severe retinoid related side-effects. Only five of the investigated patients developed mild side-effects, i.e. dry skin and lips. Even although the results come from different studies, it seems that in adults, by increasing the dose from 20 mg (equivalent to approximately 12 mg $m^{-2}$) to 5 mg $kg^{-1}$ (equivalent to approximately 185 mg $m^{-2}$), there is only a slight increase in average peaks from 1.3 to 2.5 μM. Finally, high (i.e. 7.5 μM) maximum 13*cis*RA concentrations can be achieved in children, and such concentrations are higher than those observed in adults after similar doses (i.e. 160 mg $m^{-2}$ in children and 185 mg $m^{-2}$ in adults). In some cases, we observed a secondary 13*cis*RA maximum concentration. A similar observation has been reported in healthy subjects (Khoo et al, 1982) and is consistent with enterohepatic recycling.

An average half-life of 30 h, was found in melanoma patients. Similar values were found in advanced cancer patients, in whom the half-life averaged approximately 25 h (Goodman et al, 1982). In patients suffering from cystic acne and in patients with various keratinization disorders, the half-lives were 10 and 16 h respectively (Brazzell et al, 1983), and in male volunteers the half-life was 20 h (Khoo et al, 1982). The differences between 13*cis*RA half-life in cancer and non-cancer patients might be due to differences in age.

In melanoma patients, the maximum 13*cis*RA concentrations, the AUCs and the elimination half-lives of 13*cis*RA after repeated treatments were similar on average to those of the first dose. Conversely, the AUC of the metabolite 4-oxo-13*cis*RA after repeated doses was greater than after the first dose. Thus, as reported in previous studies (Brazzell et al, 1983), the pharmacokinetics of 13*cis*RA does not change during continuous treatment. Between the first and the last 13*cis*RA dose melanoma patients received IFN-α2a associated with 13*cis*RA. Although no conclusion can be drawn, no interaction seems to occur on the pharmacokinetics of 13*cis*RA by its association with IFN-α2a, as the observed 13*cis*RA pharmacokinetics was similar to that of repeated doses of 13*cis*RA as a single agent (Brazzell et al, 1983). None of the melanoma patients had detectable levels (i.e. ≥ 5 ng $ml^{-1}$) of all-*trans*RA before or after treatment. Such findings are in contrast with previous results in advanced cancer patients treated with high doses of 13*cis*RA (3, 4 and 5 mg $kg^{-1}$) (Goodman et al, 1982). In these patients, all-*trans*RA was detected in the plasma of most patients, and its concentrations varied from 0% to 30% of the 13*cis*RA concentrations.

Analysis of the influence of 13*cis*RA on plasma retinol demonstrated that the retinoid causes a temporary reduction in retinol concentration. After the doses of 20 and 40 mg, the reduction was very slight. Baseline values were reduced on average by only 20%. However, such a reduction was associated with a statistically significant regression of retinol levels in all the patients except the one who had the lowest 13*cis*RA peak levels. The influence of 13*cis*RA on retinol plasma levels is confirmed by the correlation found between 13*cis*RA $C_{max}$ and the amount of retinol reduction. The results are in agreement with previous observations of decreased plasma retinol concentration in the rat after treatment with 13*cis*RA (Berni et al, 1993; Collins et al, 1994) and with other retinoids with modifications in the area of the retinol hydroxyl end group, such as all-*trans*RA and fenretinide (4HPR) (Berni et al, 1993). In rats, although after administration of equimolar doses 13*cis*RA was slightly less potent than the other retinoids, it caused a remarkable and dose-dependent reduction in plasma retinol concentrations (Berni et al, 1993). Fenretinide (4HPR), which is less toxic than 13*cis*RA and is administered at higher doses, causes a reduction in retinol plasma levels in humans that is proportional to the dose (Formelli et al, 1989). In breast cancer patients, retinol levels were reduced by approximately 40% 24 h after 4HPR at the dose of 200 mg, i.e. a dose five- to ten-fold higher than that of 13*cis*RA herein investigated. Other authors have reported no changes in the plasma retinol concentration following 13*cis*RA treatment in humans (Goodman et al, 1982; Sass et al, 1995). As in such studies patients received 13*cis*RA doses similar (Sass et al, 1995) or higher (Goodman et al, 1982) than those herein investigated, a possible explanation for the discordance of the results is the time of blood sampling for retinol analysis. As we have shown,

© Cancer Research Campaign 1997

after 13*cis*RA treatment, retinol levels progressively decreased, with the maximum effect occurring shortly after the maximum 13*cis*RA concentrations. In the previously reported papers (Goodman et al, 1982; Sass et al, 1995), no indication is given on the interval between drug intake and retinol assay.

### ACKNOWLEDGEMENTS

This work was supported by a grant from the Associazione Italiana per la Ricerca sul Cancro. 13-*cis*-Retinoic acid and recombinant interferon α2A were provided at no cost by Hoffman La Roche, Basle (Switzerland). We thank Dr Carmen Pollini for statistical evaluation of the data and critical discussion of the manuscript and Laura Zanesi for secretarial assistance.

### REFERENCES

Berni R, Clerici M, Malpeli G, Cleris L and Formelli F (1993) Retinoids: in vitro interaction with retinol-binding protein and influence on plasma retinol. *FASEB J* **7**: 1179–1184

Brazzell RK, Vane FM, Ehmann CW and Colburn WA (1983) Pharmacokinetics of isotretinoin during repetitive dosing to patients. *Eur J Clin Pharmacol* **24**: 695–702

Cascinelli N, Bufalino R, Morabito A and Mackie R (1994) Results of adjuvant interferon study in WHO melanoma programme. *Lancet* **343**: 913–914

Colburn WA, Gibson DM, Wiens RE and Hanigan JJ (1983) Food increases the bioavailability of isotretinoin. *J Clin Pharmacol* **23**: 534–539

Collins MD, Tzimas G, Hummler H, Burgin H and Nau H (1994) Comparative teratology and transplacental pharmacokinetics of all-trans-retinoic acid, 13-cis-retinoic acid, and retinyl palmitate following daily administrations in rats. *Toxicol Appl Pharmacol* **127**: 132–144

Fierlbeck G, Schreiner T and Rassner G (1995) Combination of highly purified human leukocyte interferon α and 13-cis-retinoic acid for the treatment of metastatic melanoma. *Cancer Immunol Immunother* **40**: 157–164

Formelli F, Carsana R, Costa A, Buranelli F, Campa T, Dossena G, Magni A and Pizzichetta M (1989) Plasma retinol level reduction by the synthetic retinoid fenretinide: a one year follow-up study of breast cancer patients. *Cancer Res* **49**: 6149–6152

Goodman GE, Einspahr JG, Alberts DS, Davis TP, Leigh SA, Chen HSG and Meyskens FL (1982) Pharmacokinetics of 13-cis-retinoic acid in patients with advanced cancer. *Cancer Res* **42**: 2087–2091

Hong WK and Itri LM (1994) Retinoids and human cancer. In *The Retinoids: Biology, Chemistry and Medicine*. Sporn MB, Roberts AB and Goodman DS (eds), pp. 597–630. Raven Press: New York

Khoo KC, Reik D and Colburn WA (1982) Pharmacokinetics of isotretinoin following a single oral dose. *J Clin Pharmacol* **22**: 395–402

Lippman SM, Kavanagh JJ, Peredes-Espinoza M, Delgadillo-Madrueno F, Peredes-Casillas P, Hong WK, Holdener E and Krakoff IH (1992*a*) 13-cis-retinoic acid plus interferon α-2a: effective combination therapy for advanced squamous cell carcinoma of the skin. *J Natl Cancer Inst* **84**: 241–245

Lippman SM, Parkinson DR, Itri LM, Weber RS, Schantz SP, Ota DM, Schusterman MA, Krakoff IH, Gutterman JU and Hong WK (1992*b*) 13-cis-retinoic acid plus interferon α-2a: effective combination therapy for advanced squamous cell carcinoma of the skin. *J Natl Cancer Inst* **84**: 235–240

Lotan R, Dawson MI, Zou CC, Jong L, Lotan D and Zou CP (1995) Enhanced efficacy of combinations of retinoic acid- and retinoid X receptor-selective retinoids and α-interferon in inhibition of cervical carcinoma cell proliferation. *Cancer Res* **55**: 232–236

Marth C, Widschwendter M and Daxenbichler G (1993) Mechanism of synergistic action of all-trans- or 9-cis-retinoic acid and interferons in breast cancer cells. *J Steroid Biochem Mol Biol* **47**: 123–126

Sass JO, Masgrau E, Saurat J-H and Nau H (1995) Metabolism of oral 9-cis-retinoic acid in the human. Identification of 9-cis-retinoyl-β-glucuronide as urinary metabolites. *Drug Metab Disp* **23**: 887–891

Schaber B, Mayer P, Schreiner T, Rassner G and Fierlbeck G (1994) Anti-proliferative activity of natural interferon-alpha, isotretinoin and their combination varies in different human melanoma cell lines. *Melanoma Res* **4**: 321–326

Sheridan E and Hancock BW (1992) Systemic treatment of metastatic malignant melanoma. In *Diagnosis and Management of Melanoma in Clinical Practice*. Kirkham N, Cotton DWK, Lallemand RC, White JE and Rosin RD (eds) pp 135–152. Springer: London

Triozzi PL, Walker MJ, Pellegrini AE and Dayton MA (1996) Isotretinoin and recombinant interferon alfa-2a therapy of metastatic malignant melanoma. *Cancer Invest* **14**: 293–298

Villablanca JG, Khan AA, Avramis VI, Seeger RC, Matthay KK, Ramsay NKC and Reynolds CP (1995) Phase I trial of 13-cis-retinoic acid in children with neuroblastoma following bone marrow transplantation. *J Clin Oncol* **13**: 894–901

© Cancer Research Campaign 1997